# Does Deep Neuromuscular Blockade Improve Operating Conditions during Minimally Invasive Anterolateral Total Hip Replacements?: A Randomized Controlled Trial

**DOI:** 10.7759/cureus.10328

**Published:** 2020-09-09

**Authors:** Craig Curry, Kyle Steen, Wendy Craig, Christopher W Cary, Janelle Richard, George Babikian

**Affiliations:** 1 Anesthesiology and Perioperative Medicine, Maine Medical Center, Portland, USA; 2 Center for Outcomes Research and Evaluation, Maine Medical Center Research Institute, Portland, USA; 3 Orthopaedic Surgery, Maine Medical Center, Portland, USA

**Keywords:** total hip replacement, neuromuscular blockade

## Abstract

Background

Neuromuscular blockade (NMB) is thought to improve operative conditions during certain procedures. Published descriptions of minimally invasive hip replacement techniques specify the need for “excellent relaxation”, however, the optimal degree of NMB required for such cases has not been studied. We performed a randomized, single-blind study comparing the effect of moderate and deep neuromuscular blockade on surgical conditions and operating time during minimally invasive anterolateral hip replacement. Vecuronium was administered to maintain moderate NMB (train-of-four count of 1-2) or deep NMB (train-of-four count of 0, post-tetanic count of 1-2).

Methods

In this study, 116 patients were randomized to receive moderate or deep neuromuscular blockade; depth of blockade was monitored using acceleromyography. The primary outcome was the number of requests from the surgeon for additional blockade intraoperatively. Secondary outcomes included operative times and assessment of the operative conditions by the surgeon utilizing the Leiden-Surgical Rating Scale.

Results

Cases with additional requests for blockade did not differ between the deep and moderate NMB groups (11/58, 19.0% vs 8/58, 13.8%); relative risk, 1.22 (95% CI [confidence interval], 0.70-2.15), p=0.62. Neither time from incision to prosthesis reduction (33.8±1.2 min vs. 32.6 ±1.2 min; difference in geometric mean, 0.96 [95% CI, 0.90-1.04] minutes, p=0.33), nor the surgeon’s assessment of operative conditions (p=0.88), differed between the deep or moderate NMB groups, respectively.

Conclusions

Deep NMB did not produce significantly improved operative conditions compared with moderate NMB. Routine use of deep NMB during minimally invasive anterolateral hip arthroplasty is not supported by this study.

## Introduction

Neuromuscular blockade (NMB) is used in many surgeries, including orthopedic and intra-abdominal procedures, to facilitate surgical exposure and improve operating conditions. Unfortunately, residual effects of NMB can lead to significant respiratory and airway complications especially if it is not adequately reversed [1]. NMB depth is most commonly monitored during surgery using peripheral nerve stimulation and measuring muscle contraction in response to a train-of-four stimulation (TOF count) or post-tetanic stimulation (PTC). Moderate NMB is characterized by maintaining one to two contractions to a train of four stimulation, while deep NMB eliminates all contractions to train of four stimulation while maintaining contractions with post-tetanic stimulation [2]. Moderate NMB is felt to provide a sufficient degree of relaxation for most surgical procedures while allowing for adequate reversal using neostigmine in most circumstances. Deep NMB is more reliably reversed with sugammadex; the risk of inadequate reversal is lower than with neostigmine but it has been reported [3]. It has been hypothesized that deep NMB may improve operating conditions during intra-abdominal surgeries [4-8]; however, the preponderance of evidence does not support that intuitive premise [9]. Further studies of specific types of surgery are needed to evaluate and confirm any benefits before a broad application of deep NMB can be recommended.

Minimally invasive hip replacement techniques, which are performed through smaller incisions, are purported to result in less trauma and consequently less pain and faster recovery, and are increasingly common [10]. Due to the smaller muscular incision, they may theoretically benefit from deeper levels of NMB for optimal surgical conditions. Published descriptions of these techniques specify the need for “excellent relaxation” however the optimal degree of NMB required for such cases has not been studied [9]. 

Our aim was to compare the effect of moderate and deep NMB on surgical conditions during minimally invasive anterolateral total hip arthroplasty (ALTHA), as assessed by intra-operative requests from the surgeon for additional relaxation. To address this, we performed a randomized study to examine the effect of deep NMB (TOF count 0, PTC 1-2) versus moderate NMB (TOF count 1-2) on surgical conditions during minimally invasive ALTHA. Our primary outcome measure was the number of requests for additional relaxation during surgery and our secondary outcome measures were operating time and improved surgeon satisfaction with operating exposure. 

## Materials and methods

Ethics statement and trial registration 

The study was performed at Maine Medical Center (MMC) in Portland, Maine between July 24, 2017, and April 30, 2018. The study protocol was approved by MMC’s Institutional Review Board (IRB #00000262) and registered at clinicaltrials.gov prior to patient enrollment (NCT03219294, Principal Investigator: Dr. Craig Curry, Date of registration: July 17, 2017). All patients who participated in the study provided written informed consent. This project was financially supported by a departmental research fund and statistical assistance was supported in part by the Northern New England Clinical and Translational Research Center’s NIH grant U54GM115516.

Randomization 

The study had a single-blind randomized design with the surgeon blinded to treatment. The research navigator randomized patients 1:1 to a moderate NMB or a deep NMB using a computer generated randomization code (NQuery + nTerim 4.0, Statistical Solutions Ltd, Boston MA); the randomization scheme used mixed block sizes and was stratified by gender. The anesthesia care providers were unblinded and were responsible for the dosing of vecuronium intended to achieve the correct depth of NMB. Randomization was performed just prior to induction of anesthesia; the anesthesia care team received the randomization code from the research coordinator at induction.

Patients

**Figure 1:**
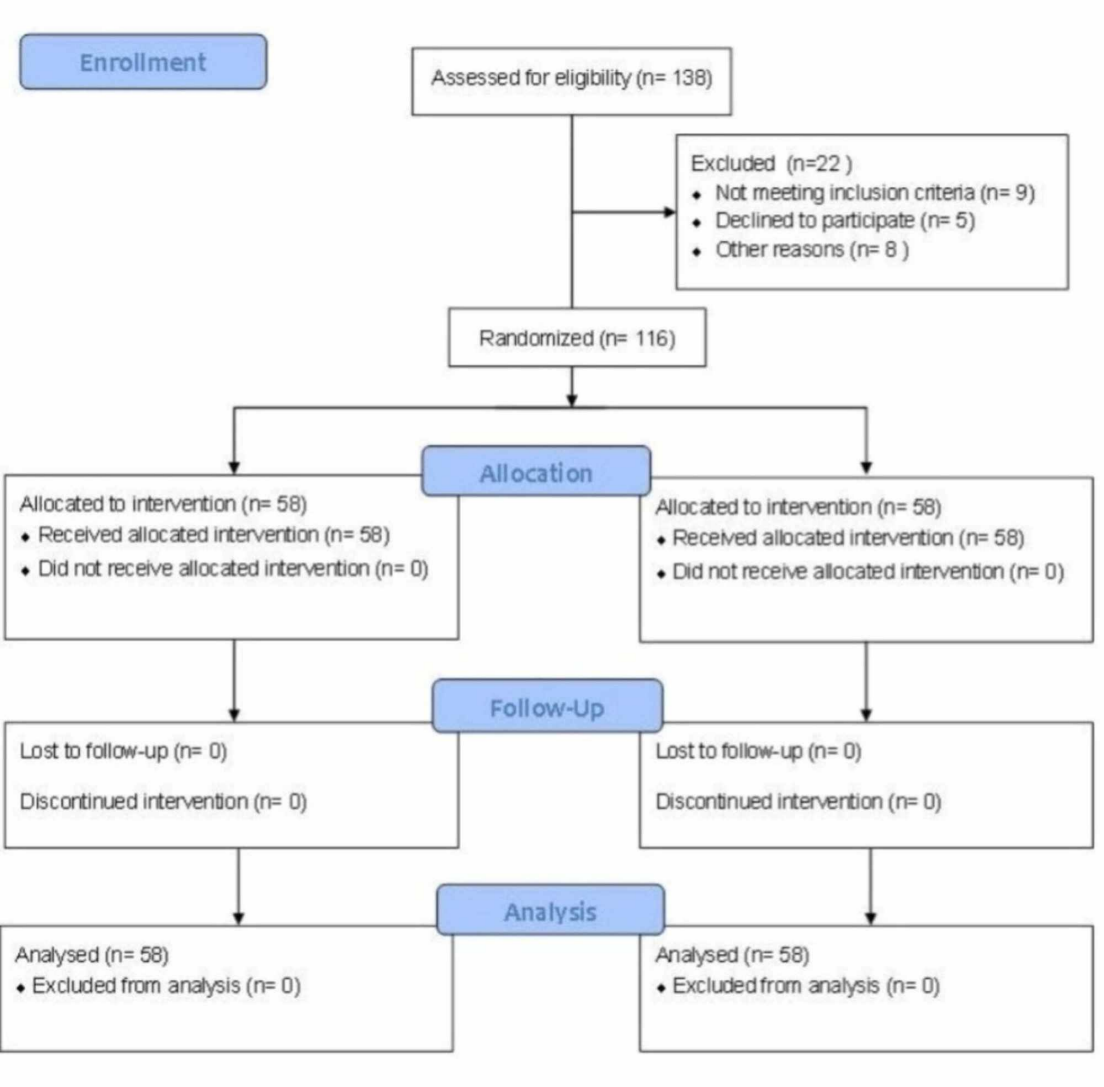
CONSORT Diagram CONSORT: Consolidated Standards of Reporting Trials

All patients aged 50 to 75 years old with an American Society of Anesthesiologists (ASA) score of 1-3 and scheduled for minimally invasive ALTHA by a single surgeon between July 24, 2017 and April 30, 2018 were potentially eligible to participate in the study. Those with BMI > 30 kg/m2, renal insufficiency, allergies to study drugs and/or contraindications to general anesthesia or NMB were excluded. Eligible patients were approached about the study sequentially, based on their scheduled surgery date, by the surgeon’s physician’s assistants. We enrolled 58 patients into each of the moderate and deep NMB groups, as shown in Figure 1. 

Monitoring and study design

All patients received pretreatment with gabapentin and acetaminophen and were monitored with automated blood pressure cuffs, pulse oximeters, and electrocardiograms (ECG). General anesthesia was induced with propofol and fentanyl and maintained with sevoflurane and intermittent doses of hydromorphone. Vecuronium was administered at induction to facilitate intubation and subsequently administered to maintain desired levels of NMB. Neuromuscular function was monitored using acceleromyography with the TOF-watch-SX monitor (MSD BV, Haarlem, The Netherlands). Two electrodes were placed over the ulnar nerve of the wrist opposite the side of replacement and additionally secured with tape. The thumb was placed in a flexible adaptor, which was also secured with tape, to generate preload and a sensor was placed on the tip of the thumb. Adduction of the thumb through contractions of the adductor pollicis muscle was detected by the sensor on the thumb. After induction of anesthesia but prior to any administration of vecuronium, the device was calibrated according to the specification of the manufacturer with the necessary modification of change in position after calibration required due to the lateral position of surgery. In the study, we measured TOF count at five-minute intervals. In the event that zero thumb twitches were detected, the PTC was measured.

In the moderate NMB group, the goal was to provide a TOF count with one to two twitches. NMB was induced at induction with a dose of vecuronium of 0.1 mg/kg using ideal body weight followed by intermittent doses of 0.0125 to 0.05 mg/kg. 

In the deep NMB group, the goal was to achieve and maintain no twitches in the TOF count, and one to two twitches in the PTC. NMB was induced at induction with a dose of vecuronium of 0.2 mg/kg ideal body weight, followed by intermittent doses of .025-0.1 mg/kg. 

If the surgeon requested deeper relaxation during surgery, this request was recorded, and if the patient was in the moderate NMB group additional vecuronium was given to achieve the target of deep NMB for the remainder of the surgery; if the patient was already in the deep NMB group, a dose of saline was administered. Doses required to maintain the target depth of NMB were recorded and additional doses requested by the surgeon due to inadequate surgical conditions were recorded separately.

On completion of closure, moderate NMB was reversed with sugammadex 2 mg/kg IBW, while deep NMB was reversed with sugammadex 4 mg/kg IBW.

Surgical technique

A standard anterolateral, minimally invasive, muscle-sparing non-cemented total hip replacement was performed by a single surgeon and surgical team using the same technique described elsewhere [11].

Data collection

We collected variables needed to assess patient eligibility (age, weight, height, sex, and ASA classification), anesthesia-related variables (vecuronium dosages, time from closure until extubation), and surgery-related variables (postoperative modified Leiden-Surgical Rating Scale, duration of surgery) from the electronic medical record. Duration of surgery was measured as the time from incision to reduction of the prosthesis; this is the time during which muscular interference with operative conditions could occur and we anticipated it to be the surgical time most likely to be affected by muscle relaxation. We also collected time from reduction of the prosthesis to extubation as an indicator of prolonged time to adequate reversal. We assessed surgeon satisfaction with operative conditions using the Leiden-Surgical Rating Scale; we modified the scale for the current orthopedic procedure as it was originally developed to evaluate surgical conditions in laparoscopic procedures (Table 1) [5]. This instrument uses a Likert scale ranging from 1 to 5: 1, extremely poor conditions; 2, poor conditions; 3, acceptable conditions; 4, good conditions; and 5, optimal conditions. The surgeon was asked to complete the scale at the conclusion of each surgery to assess their perception of overall surgical conditions. Study data were entered into a REDCap database, supported by a National Institute of Health grant UL1TR001064, prior to analysis.

**Table 1:** Modified Leiden-Surgical Rating Scale used for post-operative assessment of surgical conditions.

Likert Scale	Perceived Surgical Condition	Qualities assessed
1	Extremely poor	Muscles resistant to retraction and obscure view, implant difficult to insert into socket, requires assist
2	Poor	Muscles resistant to retraction and obscure view, implant difficult to insert into socket not requiring assist
3	Acceptable	Muscles resistant to retraction, adequate view, difficult to insert not requiring assist
4	Good	Muscles relaxed adequate view, Easy to insert
5	Optimal	Muscles relaxed excellent view, Easy to insert

Statistical analysis

Data are presented using standard descriptive statistics. Differences between treatment groups were evaluated on an intention to treat basis, using t-tests or the Mann Whitney U test for continuous variables, and chi-squared test with continuity correction, or Fisher's exact test for categorical variables, as appropriate. When continuous data were not normally distributed, data were log-transformed prior to t-tests and summary data were expressed as the geometric mean. The effect size for differences in baseline demographic data between randomization groups was calculated as standardized difference (Cohen’s d) for a difference in means and as Cohen’s h for a difference in proportions, as appropriate. Relative risk was calculated for binary categorical variables; surgical rating was converted to a binary variable by stratifying responses as good/optimal versus all other ratings. All analyses were performed using SPSS Statistical Software, version 24 (IBM SPSS Inc, Armonk, NY).

Sample size calculations estimated that a study size of n=116 (n=58 in each of the moderate and deep NMB groups) would detect a 50% reduction in the frequency of cases with one or more requests for additional NMB in the deep versus moderate NMB group, at alpha = 0.05 and 80% power. The calculations, performed using NQuery software (Statistical Solutions Ltd, Boston, MA), were based on a chi-squared test, comparing the current frequency of cases with surgeon requests for additional NMB during these surgeries at our institution (preliminary estimate, 50% of cases) and an anticipated effect size of 50% (absolute difference of 25% in the frequency of cases with additional requests for additional NMB in the deep NMB group). Since no prior studies have addressed this question among arthroplasty procedures, and no prior data are available for our binary primary outcome measure, this estimate of a 25% absolute difference in frequency was informed by clinical experience and by the data of Martini et al., who compared surgical conditions during laparoscopic surgery in patients with moderate vs. deep NMB and reported a 20% absolute difference in median surgical rating scale score between the two groups [7].

## Results

Study group characteristics are shown in Table 2. The two randomization groups were balanced for age, sex, body mass index (BMI) and ideal body weight (Cohen’s d≤0.2). No patients in either group required additional neuromuscular reversal or postoperative intubation. 

**Table 2:** Characteristics of the study group, both overall and after stratification by randomization group ^1^The effect size was calculated as Cohen’s d (continuous variables) or Cohen’s h (proportions). Cohen’s d was calculated as d=((M1-M2)/pooled standard deviation), where M1 and M2 are the means of a given variable in the moderate and deep NMB groups, respectively and pooled standard deviation (SD) is √[(SD1)2 + (SD2)2)/2]. Cohen’s h was calculated as h= [(2arcsin√P1) - (2 arcsin√P2)], where P1 and P2 are the proportion of the two groups. Effect size ≤0.2 was interpreted as a small effect size.

	Mean (SD) or n (%)		Effect Size^1^
NMB	Moderate	Deep	
n	58	58	
Male sex	29 (50.0)	28 (48.3)	0.034
Female sex	29 (50.0)	30 (51.7)	0.035
Age (years)	63.1 (6.7)	62.9 (7.4)	0.028
Body mass index (kg/m^2^)	27.5 (3.7)	27.1 (3.7)	0.108
Ideal body weight (kg)	65.7 (10.7)	65.5 (9.3)	0.020

Table 3 shows operative data for the two NMB groups. Patients in the deep NMB group received a significantly larger dose of vecuronium (p<0.001), even after responding to surgeon requests for additional NMB in the moderate group. 

**Table 3:** Operative findings ^1^Data condensed as none/any request and analyzed by chi square test with continuity correction ^2^Mann Whitney U test ^3^Data are shown as geometric mean± standard deviation; data were log transformed prior to t test and the difference represents the difference in geometric means (95% confidence interval). ^4^t test SD: standard deviation; NMB: neuromuscular blockade; CI: confidence interval

	Mean (SD), Median [range] or Frequency, n (%)				
	NMB				
Variable	Deep	Moderate	Relative risk	Mean difference	p-value
			(Deep vs Moderate)	(Deep - Moderate)	
			(95% CI)	(95% CI)	
Primary outcome					
Surgeries with additional requests for NMB	11/58 (19.0)	8/58 (13.8)	1.22 (0.70-2.15)		0.62^1^
Secondary Outcomes					
Postoperative rating of surgical conditions					
Extremely poor	2 (3.4)	1 (1.7)			0.88^2^
Poor	10 (17.2)	11 (19.0)			
Acceptable	29 (50.0)	30 (51.7)			
Good	15 (25.9)	15 (25.9)			
Optimal	2 (3.4)	1 (1.7)			
Operative time (minutes)^3^					
Incision to prosthesis reduction	33.8 (1.2)	32.6 (1.2)		0.96 (0.90-1.04)	0.33^4^
Prosthesis reduction to extubation	24.0 (1.2)	23.0 (1.2)		1.04 (0.90-1.24)	0.21^4^
Intervention					
Vecuronium dose (mg)	14.1 (3.3)	9.2 (2.5)		4.85 (3.77-5.93)	<0.001^4^

The surgeon asked for additional muscle relaxation in 19 of 116 cases (16%). There was no significant difference in the frequency of requests for additional relaxation in the deep (11/58, 19%) and moderate (8/58, 13.8%) NMB groups, relative risk, 1.22 (95% CI, 0.70-2.15), p=0.62). There was also no difference in the distribution of the surgeon’s operative condition rating between the two groups (p=0.88); the frequency of good/optimal ratings was 17/58, (29.3%) in the deep NMB group and 16/58 (27.6%) in the moderate NMB group.

There were no significant differences between deep and moderate NMB groups in the times from incision to prosthesis reduction (difference in geometric mean, 0.96 (95% CI, 0.90-1.04) minutes, p=0.33), as well as the time from prosthesis reduction to extubation (difference in geometric mean, 1.04 (0.90-1.02) minutes, p=0.21). 

## Discussion

In the present study, the first to look specifically at minimally invasive ALTHA performed under general anesthesia, we did not find evidence of reduced requests for additional relaxation with deep NMB. We also did not find an improved perception of operating conditions nor a reduction in operating time among patients given deep versus moderate NMB. 

While it seems intuitive that muscle relaxation would improve operative conditions, the findings of studies investigating this have been inconsistent. Most such studies have focused on intra-abdominal and retroperitoneal procedures and while some report that deep NMB is associated with improved operating conditions for the surgeon [4-8], a systematic review has highlighted limitations among published studies and comments on potential economic repercussions of deeper NMB [9].

Nevertheless, it is common practice for orthopedic surgeons at our institution to request additional relaxation when operating conditions are perceived to be difficult; however, we could find no prior study of the impact of depth of NMB in orthopedic operations. In the present study, the first to look specifically at minimally invasive ALTHA performed under general anesthesia, we did not find evidence of reduced requests for additional relaxation with deep NMB. We also did not find an improved perception of operating conditions nor a reduction in operating time among patients given deep versus moderate NMB.

The lack of benefit we observed may be characteristic of minimally invasive ALTHA and does not necessarily apply to other total hip arthroplasty techniques. Factors other than depth of NMB may be more important determinants of surgical conditions in minimally invasive ALTHA. It may be that restricted joint mobility related to stiffer ligaments and tendons in older patients requiring minimally invasive ALTHA may play a greater role in producing difficult operating conditions than incompletely relaxed muscles. Perhaps in a surgical population such as young and muscular or obese populations, deep NMB may have benefits. The surgeon in this study did request additional muscle relaxation in 16% of cases without a significant difference between deep and moderately blocked patients. This does suggest that he was attributing difficulty with exposure to inadequate relaxation incorrectly. Other factors such as ligamentous and tendon stiffening likely contribute to difficulty with exposure and are not affected by increasing depth of muscle relaxation. Other surgeons may be more or less likely to correctly attribute the difficulty from such factors and request additional relaxation more or less often. 

Another potential explanation for the observed lack of benefit from deep NMB in this patient population may be differential effects of NMB on different muscle groups in the body. One recent study which examined the rocuronium dose required to block direct obturator nerve stimulation of adductor muscles of the thigh found that complete blockade of the thigh adductors was achieved before TOF count responses in the adductor pollicis were suppressed [12]. We measured depth of NMB at the adductor pollicis and thus cannot rule out the possibility that the response of this muscle to NMB may have differed to the response of muscles, affecting the operative site during minimally invasive ALTHA. If hip girdle muscles are more sensitive to NMB than the adductor pollicis, this would suggest that patients with moderate depth of NMB as measured at adductor pollicis may have had deep NMB at the operative site and this would minimize the difference in depth of NMB at the operative site that was suggested by adductor pollicis monitoring.

Our study has several limitations. First, we utilized a single surgeon, which may limit the generalizability of our findings. This was done to reduce variability in assessing operating conditions and thus increase the likelihood of observing a difference. The particular surgeon who was chosen performs a high volume of minimally invasive ALTHA and specifically requests deep NMB during surgery which we felt increased the chance of finding any possible advantage of deep NMB. Second, the scale we used to measure surgeon satisfaction with operating conditions was modified from the Leiden-Surgical Rating Scale, which has been utilized to assess NMB effects for laparoscopic intra-abdominal surgery [7]. While the surgeon in that study was able to discriminate deep from moderate degrees of relaxation using that scale, there has been no other validation of such scales for the study of muscle relaxation and specifically, there has not been any validation in orthopedic surgery. If this scale lacked sensitivity this may explain the lack of effect measured by this scale. Third, we did not measure consistency with achieving the intended level of NMB. The larger doses of veruconium in the deep NMB group suggest that our intervention was effective overall; however, it may not have been entirely consistent throughout. Finally, the observed rate of requests for additional relaxation (13.8-19%) was lower than the estimate of 50%, based on pre-study experience, that we used in our original power analysis. This change may reflect a Hawthorne effect on the part of our surgeon or possibly deeper planes of relaxation in even the moderate NMB group than had been provided before the study as we do not routinely utilize accelomygraphy in our clinical practice and thus may have routinely provided lesser degrees of relaxation than those achieved in the present study. Our original power analysis was based on a 50% reduction in frequency of additional requests for relaxation with deep NMB; post hoc power analysis indicates that our sample size of n=116 had 14% power at alpha = 0.05 to detect a 50% reduction from the 13.8% frequency actually observed in the moderate NMB group. Despite reduced power to detect a statistical difference between groups, the observed lack of effect of deep NMB on the frequency of requests for further relaxation indicates that deep NMB is still unlikely to confer a clinically significant advantage.

Deep NMB requires a larger dose of NMB and larger doses of either neostigmine or sugammadex for adequate reversal. Reversal from greater depths of relaxation does increase the risk of less than optimal reversal with either neostigmine or sugammadex [13]. Using sugammadex may also inhibit the ability to re-paralyze patients, should that be required in the immediate postoperative period, and possibly induce serious bradycardias and hypotension [3,14]. Finally, the larger dose of sugammadex required to reverse deep NMB will increase the direct cost of care [15]. Thus there should be demonstrable benefits of deep NMB to justify the increased expense of achieving and reversing deep NMB and the increased risk, albeit small, of inadequate reversal before deep NMB is routinely applied in any surgical population. 

## Conclusions

In summary, we compared operating conditions during ALTHA under moderate and deep NMB conditions. We did not find evidence that deep NMB improved surgeon perceptions of operating conditions nor did it reduce operating time during minimally invasive ALTHA compared to moderate NMB conditions. Deep NMB has increased direct expenses and increased risks including respiratory insufficiency. The need for NMB varies by type of surgery; there should be clear evidence of benefit for specific types of surgery before deep NMB is utilized routinely in these cases. The results of this study do not support routine use of deep NMB for minimally invasive ALTHA and are consistent with the lack of additional benefits seen in laparoscopic surgeries.
